# Supplementary material for: Quantification of Anopheles daily sugar feeding rates in Siaya county, western Kenya using Attractive Sugar Baits
Source: PLoS One. 2025 Nov 24;20(11):e0337207. doi: 10.1371/journal.pone.0337207 (PMC12643295; doi:10.1371/journal.pone.0337207)
Supplement: S2 Fig — The upper and lower bounderies of the error bars represent the 95% confidence interval and ‘NS’ if not significant. (DOCX) [file pone.0337207.s002.docx]

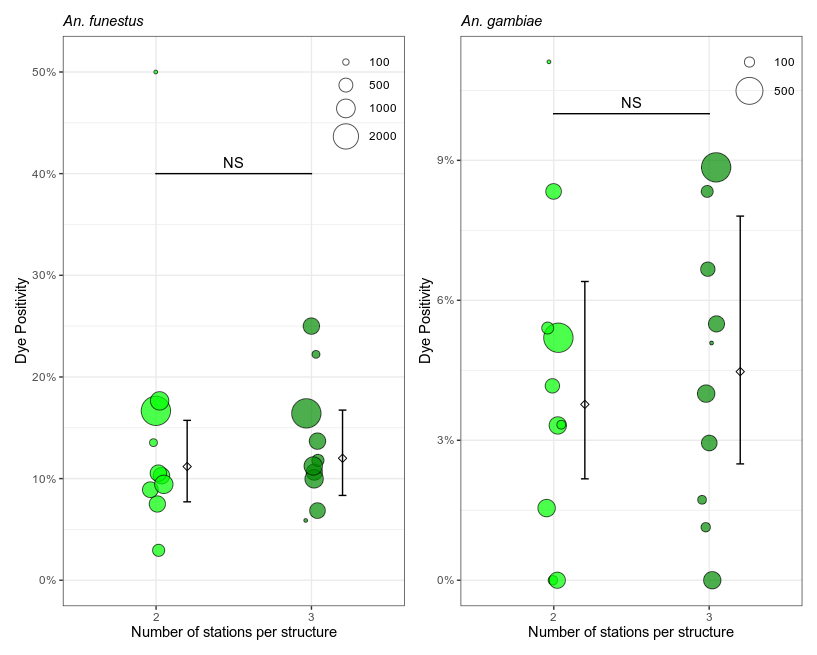


S2 Fig: Comparison of the impact of 2 versus 3 bait stations on *An. funestus* and *An. gambiae* dye positivity. The upper and lower bounderies of the error bars represent the 95% confidence interval and ‘NS’ if not significant
