# Supplementary material for: Quantification of Anopheles daily sugar feeding rates in Siaya county, western Kenya using Attractive Sugar Baits
Source: PLoS One. 2025 Nov 24;20(11):e0337207. doi: 10.1371/journal.pone.0337207 (PMC12643295; doi:10.1371/journal.pone.0337207)
Supplement: S3 Fig — P-value significance is indicated by ‘*’ if ≤ 0.05, ‘***’ if ≤ 0.001 with the upper and lower bounderies indicating the 95% confidence interval and ‘NS’ if not significant. (DOCX) [file pone.0337207.s003.docx]

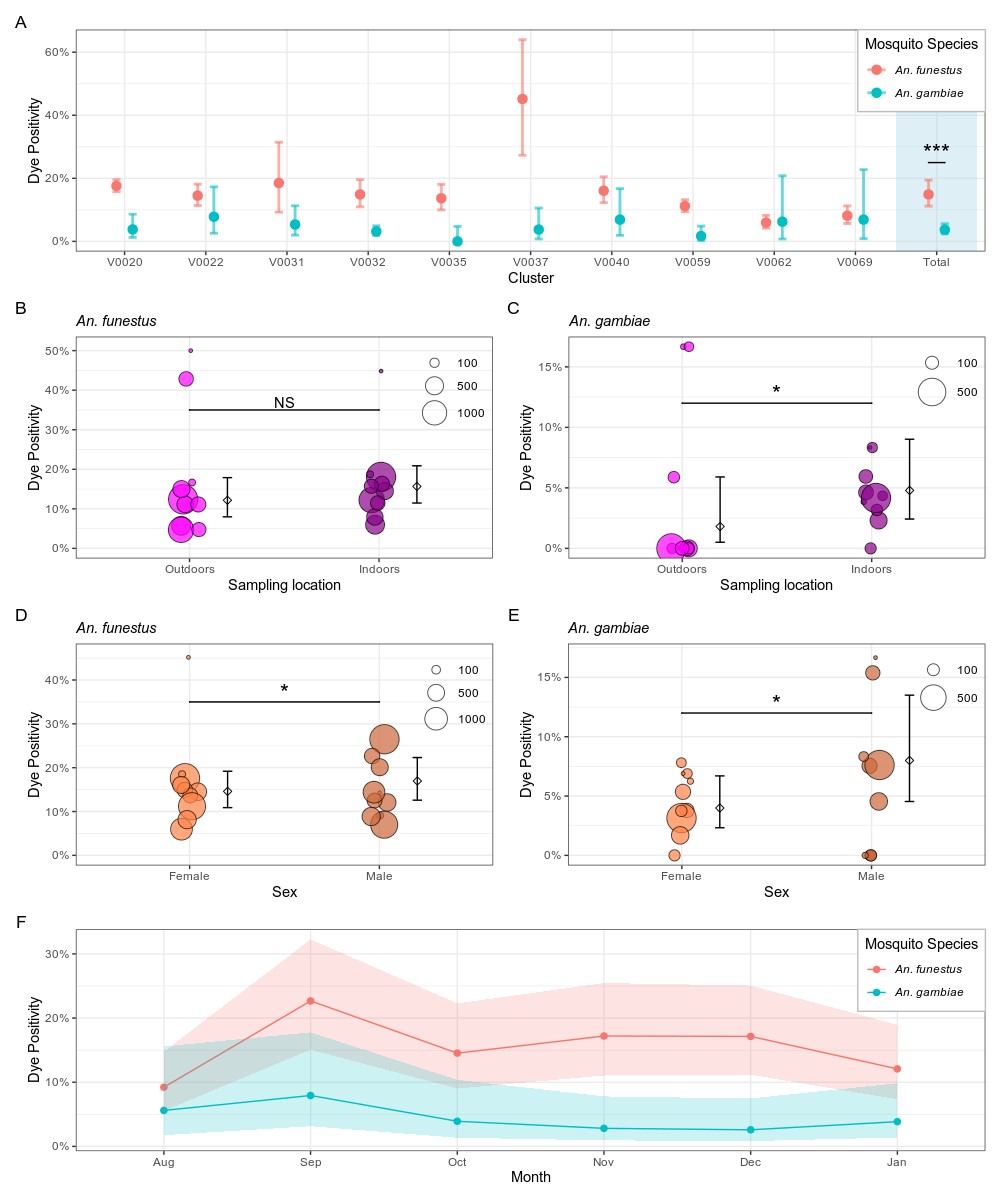


**S3 Fig**: Comparison of percentage dye positivity of *Anopheles* mosquitoes by; cluster (A), indoor and outdoor sampling locations for *An. funestus* (B) and *An. gambiae* (C), sex for *An. funestus* (D) and *An. gambiae* (E) and collection month (F). P-value significance is indicated by ‘*’ if ≤ 0.05, ‘***’ if ≤ 0.001 with the upper and lower bounderies indicating the 95% confidence interval and ‘NS’ if not significant.
