# Supplementary material for: Quantification of Anopheles daily sugar feeding rates in Siaya county, western Kenya using Attractive Sugar Baits
Source: PLoS One. 2025 Nov 24;20(11):e0337207. doi: 10.1371/journal.pone.0337207 (PMC12643295; doi:10.1371/journal.pone.0337207)
Supplement: S4 Fig — The middle dashed black line displays the estimated DFR required to achieve a 30% reduction with the upper and lower bounderies indicating the 95% confidence interval of this estimate. (DOCX) [file pone.0337207.s004.docx]

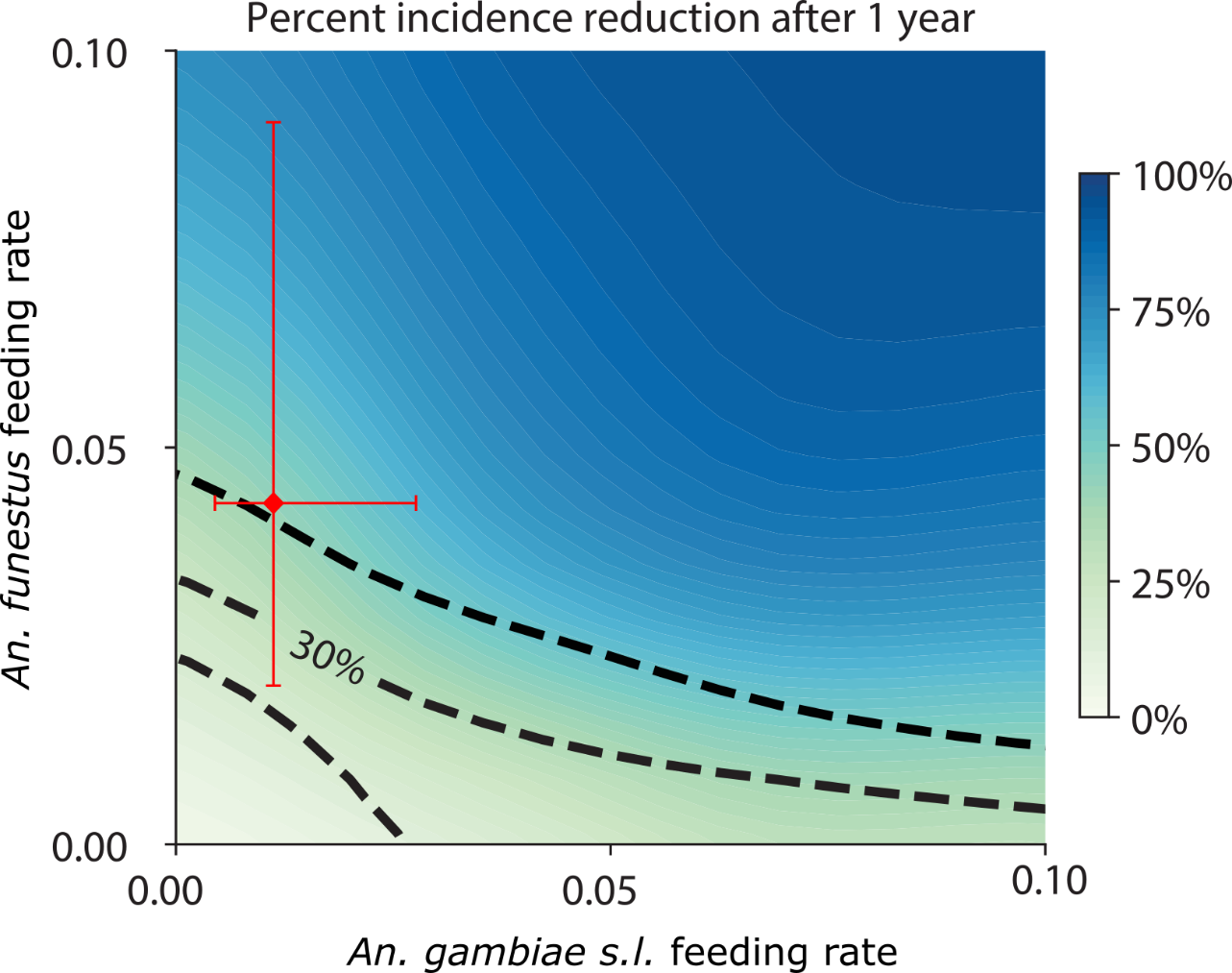


**S4 Fig:** Estimation of daily feeding rate (DFR) generated from the study derived dye positivity (red diamond) with the associated 95% confidence interval overlaid on the estimated percentage reduction in malaria incidence in one year in western Kenya for a given *An. funestus* and *An. gambiae* DFR. The middle dashed black line displays the estimated DFR required to achieve a 30% reduction with the upper and lower bounderies indicating the 95% confidence interval of this estimate.
