# Supplementary material for: Quantification of Anopheles daily sugar feeding rates in Siaya county, western Kenya using Attractive Sugar Baits
Source: PLoS One. 2025 Nov 24;20(11):e0337207. doi: 10.1371/journal.pone.0337207 (PMC12643295; doi:10.1371/journal.pone.0337207)
Supplement: S5 Fig — The crossover trial period is highlighted by “Pre” (pink shading), “Post” (blue shading), and “Extended” (green shading). Horizontal coloured dashed lines indicate the mean weekly rainfall for the associated colour period. (DOCX) [file pone.0337207.s005.docx]

**
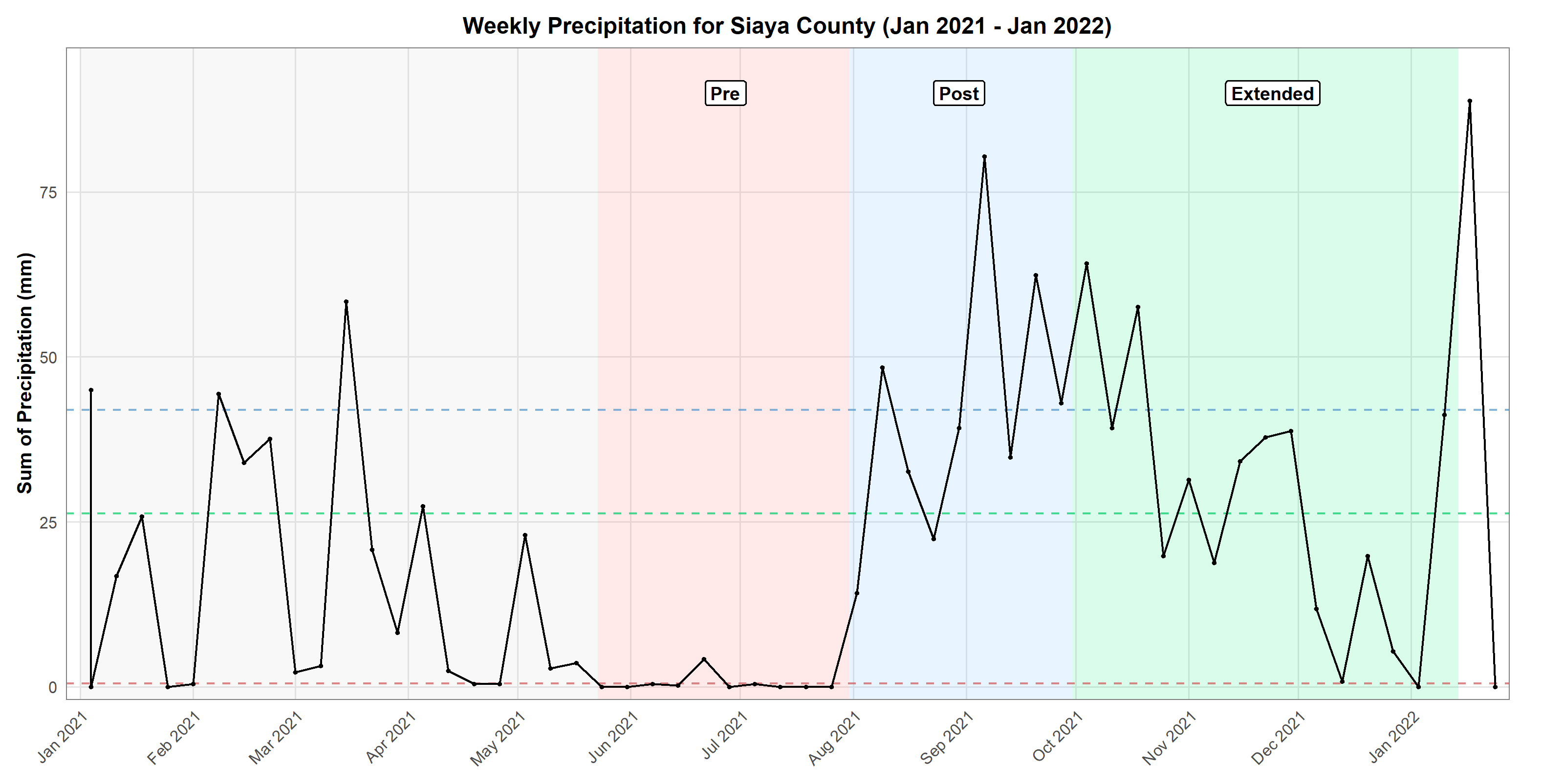
**

**S5 Fig:** Weekly precipitation (mm) for Siaya County (January 2021–January 2022). The crossover trial period is highlighted by "Pre" (pink shading), "Post" (blue shading), and "Extended" (green shading). Horizontal coloured dashed lines indicate the mean weekly rainfall for the associated colour period.
