# Supplementary material for: Quantification of Anopheles daily sugar feeding rates in Siaya county, western Kenya using Attractive Sugar Baits
Source: PLoS One. 2025 Nov 24;20(11):e0337207. doi: 10.1371/journal.pone.0337207 (PMC12643295; doi:10.1371/journal.pone.0337207)
Supplement: S1 Table — (DOCX) [file pone.0337207.s006.docx]

**S1 Table:** Number of ASB stations and structure allocations during pre and post crossover period

|  |  |  | Pre-crossover period | | |  |  | Post-crossover period | | |  |
| --- | --- | --- | --- | --- | --- | --- | --- | --- | --- | --- | --- |
| Village | Village Code | # of Structures with ASBs | | # of Stations Deployed/ Structure* | Total # of ASBs Deployed | | # of Structures with ASBs | | # of Stations Deployed/ Structure* | Total # of ASBs Deployed | |
| Abwao | 20 | 465 | | 2 | 932 | | 534 | | 3 | 1602 | |
| Akom | 32 | 381 | | 2 | 762 | | 421 | | 3 | 1263 | |
| Kaonje | 22 | 362 | | 2 | 724 | | 360 | | 3 | 1080 | |
| Nyore | 31 | 439 | | 2 | 878 | | 490 | | 3 | 1470 | |
| Ombulu Masanga | 59 | 273 | | 2 | 546 | | 269 | | 3 | 807 | |
| Kawino | 40 | 353 | | 3 | 1059 | | 366 | | 2 | 732 | |
| Kitambo | 69 | 571 | | 3 | 1713 | | 546 | | 2 | 1092 | |
| Konge | 62 | 655 | | 3 | 1965 | | 653 | | 2 | 1306 | |
| Rakombe | 37 | 502 | | 3 | 1506 | | 481 | | 2 | 962 | |
| Sinogo | 35 | 313 | | 3 | 939 | | 314 | | 2 | 628 | |
| Saradidi/Miyare** | 24 | 0 | | 0 | 0 | | 0 | | 0 | 0 | |
| Jusa** | 33 | 0 | | 0 | 0 | | 0 | | 0 | 0 | |
| **Total** |  | **4314** | |  | **11024** | | **4434** | |  | **10942** | |

*Targeted number of bait stations per structure

**Control villages
