# Supplementary material for: Quantification of Anopheles daily sugar feeding rates in Siaya county, western Kenya using Attractive Sugar Baits
Source: PLoS One. 2025 Nov 24;20(11):e0337207. doi: 10.1371/journal.pone.0337207 (PMC12643295; doi:10.1371/journal.pone.0337207)
Supplement: S2 Table — (DOCX) [file pone.0337207.s007.docx]

S2 Table: Bait station density by cluster

|  |  |  | Pre-Crossover | | | |  |  | Post-Crossover | | | |  |
| --- | --- | --- | --- | --- | --- | --- | --- | --- | --- | --- | --- | --- | --- |
| Cluster | Density Type | ASBs deployed | | Structure Density/Ha | Bait Density | Stations per structure | | ASBs deployed | | Structure Density/Ha | Bait Density | Stations per structure | |
| Abwao | Density | 2 | | 6.01 | 11.31 | 1.88 | | 3 | | 6.01 | 17.64 | 2.92 | |
|  | Weighted density |  | | 8.88 | 16.36 |  | |  | | 8.88 | 24.82 |  | |
| Akom | Density | 2 | | 4.85 | 8.34 | 1.72 | | 3 | | 4.85 | 12.97 | 2.64 | |
|  | Weighted density |  | | 7.50 | 13.04 |  | |  | | 7.50 | 19.54 |  | |
| Kaonje | Density | 2 | | 3.88 | 7.70 | 1.99 | | 3 | | 3.88 | 10.99 | 2.77 | |
|  | Weighted density |  | | 5.82 | 11.46 |  | |  | | 5.82 | 16.79 |  | |
| Nyore | Density | 2 | | 4.96 | 9.23 | 1.86 | | 3 | | 4.96 | 13.22 | 2.66 | |
|  | Weighted density |  | | 7.43 | 13.74 |  | |  | | 7.43 | 20.14 |  | |
| Ombulu Masanga | Density | 2 | | 6.32 | 11.25 | 1.78 | | 3 | | 6.32 | 16.85 | 2.65 | |
|  | Weighted density |  | | 8.93 | 16.21 |  | |  | | 8.93 | 23.16 |  | |
| Kawino | Density | 3 | | 4.94 | 13.01 | 2.63 | | 2 | | 4.94 | 9.00 | 1.75 | |
|  | Weighted density |  | | 7.05 | 20.40 |  | |  | | 7.05 | 13.37 |  | |
| Kitambo | Density | 3 | | 5.34 | 14.64 | 2.74 | | 2 | | 5.34 | 9.32 | 1.73 | |
|  | Weighted density |  | | 8.55 | 22.93 |  | |  | | 8.55 | 14.63 |  | |
| Sinogo | Density | 3 | | 4.51 | 12.85 | 2.85 | | 2 | | 4.51 | 8.83 | 1.94 | |
|  | Weighted density |  | | 6.70 | 19.35 |  | |  | | 6.70 | 13.01 |  | |
| Rakombe | Density | 3 | | 5.97 | 16.47 | 2.76 | | 2 | | 5.97 | 10.73 | 1.69 | |
|  | Weighted density |  | | 9.72 | 25.45 |  | |  | | 9.72 | 16.41 |  | |
