# Supplementary material for: Quantification of Anopheles daily sugar feeding rates in Siaya county, western Kenya using Attractive Sugar Baits
Source: PLoS One. 2025 Nov 24;20(11):e0337207. doi: 10.1371/journal.pone.0337207 (PMC12643295; doi:10.1371/journal.pone.0337207)
Supplement: Attachment — Submitted filename: Reviewer comments_Jackline et al. 2025.docx [file pone.0337207.s008.docx]

**My Comments:**

**General:** The manuscript is well-structured, scientifically rigorous, and highly relevant to the field of malaria vector control as it reports a robust field study that evaluates the sugar feeding behavior of malaria vectors using Attractive Sugar Baits (ASBs) in western Kenya. The research fills an important gap in understanding vector behavior in regions with abundant natural sugar sources and informs deployment strategies for ATSBs in subsequent large-scale trials. I have some questions and suggestions below:

1. Lines 85–86: The statement 'Attractive targeted sugar baits (ATSBs) are a candidate vector control tool that takes advantage of mosquito sugar feeding behavior' feels incomplete. I suggest expanding it to include a brief explanation of how ATSBs/ASBs work—specifically, how they attract mosquitoes and deliver a toxicant through sugar ingestion—as part of the introduction.
2. Lines 286–295: ASB stations were monitored and replaced throughout the study. This is a key aspect of the study design, and it would be valuable to present this information in table form, showing the durability of the stations over the entire study period. Durability is a standard parameter in ITN bioefficacy monitoring and should likewise be reported for ASB stations.
3. Lines 324–325 state that “significantly higher dye positivity rates were observed in males compared to females for both An. funestus and An. gambiae.” However, since **only female *Anopheles* mosquitoes transmit malaria**, the **implication of higher male feeding rates on the overall effectiveness of ASBs**—and ultimately on malaria control—remains unclear. **Please elaborate in the Discussion section** on how this finding affects the interpretation of ASB efficacy.
4. The authors reported in the Results section in line 331-334 that the overall daily feeding rate on ASBs was estimated at 4.8% for An. funestus and 1.2% for An. gambiae. However, the methods used to obtain these estimates are not described in the Methods section. Would the authors consider including a description of these methods in the Methods section?
5. The authors reported the Relationship of bait station density and rainfall to dye positivity in from line 340. These are not described anywhere in the Methods section. Would the authors please do so?
6. Sporozite detection has been mention in line 272-275. I understand that this was not the main call of this paper but it is important to include a supplementary table showing these results.
7. The conclusion in line 429 does not address the objectives of this study. Please revise it to include the key findings in relation to the stated objectives.
