# Supplementary material for: Quantification of Anopheles daily sugar feeding rates in Siaya county, western Kenya using Attractive Sugar Baits
Source: PLoS One. 2025 Nov 24;20(11):e0337207. doi: 10.1371/journal.pone.0337207 (PMC12643295; doi:10.1371/journal.pone.0337207)
Supplement: Attachment — Submitted filename: Response to reviewers_30Oct2025_Clean.docx [file pone.0337207.s010.docx]

Jackline Jeruto Kosgei

Research Scientist

Kenya Medical Research Institute (KEMRI)

P. O. Box 1578-40100,

Kisumu, Kenya

23^rd^ October 2025

James Colborn
Academic Editor
PLOS ONE

Dear Sir/Madam,

**RE; RESPONSE TO REVIEWERS**

We would like to express our sincere gratitude to you and the reviewers for the thoughtful and constructive feedback on our manuscript entitled “Quantification of Anopheles daily sugar feeding rates in Siaya County, Western Kenya using Attractive Sugar Baits” (Authored by: Jackline Kosgei, Daniel P. McDermott, Seline Omondi, Vincent Moshi, Martin J. Donnelly, Collins Ouma, Prashanth Selvaraj, Angela F. Harris, Julian Entwistle, Feiko O. ter Kuile, Keith Fraser, Lazaro Mwandigha, Bernard Abong’o, John E. Gimnig, and Eric Ochomo).

We are pleased that the reviewers and handling editor found our initial responses satisfactory and We sincerely appreciate the constructive feedback and guidance that have strengthened our manuscript. Below, we provide a detailed point-by-point response outlining the revisions made and clarifications provided.

 Journal Requirements:

When submitting your revision, we need you to address these additional requirements.

1. Please ensure that your manuscript meets PLOS ONE's style requirements, including those for file naming. The PLOS ONE style templates can be found at

https://journals.plos.org/plosone/s/file?id=wjVg/PLOSOne_formatting_sample_main_body.pdf and [https://journals.plos.org/plosone/s/file?id=ba62/PLOSOne_formatting_sample_title_authors_affiliations.pdf](https://track.editorialmanager.com/CL0/https:%2F%2Fjournals.plos.org%2Fplosone%2Fs%2Ffile%3Fid=ba62%2FPLOSOne_formatting_sample_title_authors_affiliations.pdf/1/010f0198a877b1a4-38679ca3-e103-4d0d-8625-448643e1b603-000000/pzm-eheBH3P5JRgll2k7uxiepmAxiNAEzuzY_Kk4BLI=224)

**Response:** This has been done

2. We note that the grant information you provided in the ‘Funding Information’ and ‘Financial Disclosure’ sections do not match.

When you resubmit, please ensure that you provide the correct grant numbers for the awards you received for your study in the ‘Funding Information’ section.

**Response:** We are very grateful to the editor for noting this inconsistency. We have carefully reviewed and corrected the grant details to ensure consistency between the Funding Information and Financial Disclosure sections of the submission forms.

3. Thank you for stating in your Funding Statement:

[This study was funded by IVCC through support from the Bill and Melinda Gates Foundation (INV-007509), the Swiss Agency for Development and Cooperation (SDC) (Grant: 81067480) and UK Aid (Grant: 30041-105). The findings and conclusions contained within are those of the authors and do not necessarily reflect positions or policies of IVCC, the Bill & Melinda Gates Foundation, SDC or UK Aid.].

Please provide an amended statement that declares *all* the funding or sources of support (whether external or internal to your organization) received during this study, as detailed online in our guide for authors at http://journals.plos.org/plosone/s/submit-now.  Please also include the statement “There was no additional external funding received for this study.” in your updated Funding Statement.

Please include your amended Funding Statement within your cover letter. We will change the online submission form on your behalf.

**Response**: The funding statement for the online submission has been amended as follows: “This study was funded by the Innovative Vector Control Consortium (IVCC) through support from the Bill & Melinda Gates Foundation (INV-007509), the Swiss Agency for Development and Cooperation (SDC) (Grant: 81067480), and UK Aid (Grant: 30041-105). There was no additional external funding received for this study. The findings and conclusions contained within are those of the authors and do not necessarily reflect the positions or policies of IVCC, the Bill & Melinda Gates Foundation, SDC, or UK Aid.”

4. Please expand the acronym “IVCC” (as indicated in your financial disclosure) so that it states the name of your funders in full.

This information should be included in your cover letter; we will change the online submission form on your behalf.

**Response:** The acronym IVCC stands for the Innovative Vector Control Consortium. Thank you for your accepting to revise the online submission form with this information on our behalf

5. Thank you for stating the following in the Acknowledgments Section of your manuscript:

[We also acknowledge IVCC for funding the study through support from the Bill and Melinda Gates Foundation, the Swiss Agency for Development and Cooperation (SDC) and UK Aid.]

We note that you have provided funding information that is currently declared in your Funding Statement. However, funding information should not appear in the Acknowledgments section or other areas of your manuscript. We will only publish funding information present in the Funding Statement section of the online submission form.

Please remove any funding-related text from the manuscript and let us know how you would like to update your Funding Statement. Currently, your Funding Statement reads as follows:

[This study was funded by IVCC through support from the Bill and Melinda Gates Foundation (INV-007509), the Swiss Agency for Development and Cooperation (SDC) (Grant: 81067480) and UK Aid (Grant: 30041-105). The findings and conclusions contained within are those of the authors and do not necessarily reflect positions or policies of IVCC, the Bill & Melinda Gates Foundation, SDC or UK Aid.]

Please include your amended statements within your cover letter; we will change the online submission form on your behalf.

**Response:** All funding related texts have been removed from the manuscript as advised including the acknowledgment section. The funding statement has been amended as indicated in point 3 above.

6. When completing the data availability statement of the submission form, you indicated that you will make your data available on acceptance. We strongly recommend all authors decide on a data sharing plan before acceptance, as the process can be lengthy and hold up publication timelines. Please note that, though access restrictions are acceptable now, your entire data will need to be made freely accessible if your manuscript is accepted for publication. This policy applies to all data except where public deposition would breach compliance with the protocol approved by your research ethics board. If you are unable to adhere to our open data policy, please kindly revise your statement to explain your reasoning and we will seek the editor's input on an exemption. Please be assured that, once you have provided your new statement, the assessment of your exemption will not hold up the peer review process.

**Response:** Thank you for the guidance on the data availability policy. We confirm that the data underlying this study will be made freely accessible upon acceptance of the manuscript, in accordance with PLOS ONE requirements. We are in the process of preparing the dataset and will deposit it in a publicly accessible repository prior to publication. The corresponding link/DOI will be provided once available.

7. Please amend the manuscript submission data (via Edit Submission) to include author Jackline Kosgei.

**Response:** The manuscript submission data has now been amended to include Jackline Kosgei as an author

8. Please amend your authorship list in your manuscript file to include author Jackline Jeruto Kosgei.

**Response:** The authorship list in the manuscript file has now been amended to include Jackline Jeruto Kosgei as an author **(Line 4)**

9. Please include your full ethics statement in the ‘Methods’ section of your manuscript file. In your statement, please include the full name of the IRB or ethics committee who approved or waived your study, as well as whether or not you obtained informed written or verbal consent. If consent was waived for your study, please include this information in your statement as well.

**Response:** A section on ethical considerations has been added to the methods section. The statement reads as follows:

**“Ethical Consideration:** This study was reviewed and approved by the Kenya Medical Research Institute Scientific and Ethical Review Unit (KEMRI SERU; Approval No. 3613). Additional approvals were obtained from the Liverpool School of Tropical Medicine Research Ethics Committee (Approval No. LSTM 18–015) and the U.S. Centers for Disease Control and Prevention (CDC; Approval No. 7112) under a reliance agreement with KEMRI SERU. Voluntary, informed consent was obtained from the head of each household enrolled in the study to receive sugar baits containing a marker dye. For mosquito collections, oral consent was sought from households on the day of collection before placing UV light traps. All consent procedures were conducted in the local languages (DhoLuo and Kiswahili) and back-translated into English to ensure accuracy.” **(Lines 400-408)**

10. Please include a separate caption for each figure in your manuscript.

**Response:** A separate caption has been included for each figure in the manuscript

11. We note that Supplemental Figure 1 in your submission contain map images which may be copyrighted. All PLOS content is published under the Creative Commons Attribution License (CC BY 4.0), which means that the manuscript, images, and Supporting Information files will be freely available online, and any third party is permitted to access, download, copy, distribute, and use these materials in any way, even commercially, with proper attribution. For these reasons, we cannot publish previously copyrighted maps or satellite images created using proprietary data, such as Google software (Google Maps, Street View, and Earth). For more information, see our copyright guidelines: http://journals.plos.org/plosone/s/licenses-and-copyright.

We require you to either (1) present written permission from the copyright holder to publish these figures specifically under the CC BY 4.0 license, or (2) remove the figures from your submission:

1. You may seek permission from the original copyright holder of Supplemental Figure 1 to publish the content specifically under the CC BY 4.0 license.

We recommend that you contact the original copyright holder with the Content Permission Form (http://journals.plos.org/plosone/s/file?id=7c09/content-permission-form.pdf) and the following text:

“I request permission for the open-access journal PLOS ONE to publish XXX under the Creative Commons Attribution License (CCAL) CC BY 4.0 (http://creativecommons.org/licenses/by/4.0/). Please be aware that this license allows unrestricted use and distribution, even commercially, by third parties. Please reply and provide explicit written permission to publish XXX under a CC BY license and complete the attached form.”

Please upload the completed Content Permission Form or other proof of granted permissions as an "Other" file with your submission.

In the figure caption of the copyrighted figure, please include the following text: “Reprinted from [ref] under a CC BY license, with permission from [name of publisher], original copyright [original copyright year].”

2. If you are unable to obtain permission from the original copyright holder to publish these figures under the CC BY 4.0 license or if the copyright holder’s requirements are incompatible with the CC BY 4.0 license, please either i) remove the figure or ii) supply a replacement figure that complies with the CC BY 4.0 license. Please check copyright information on all replacement figures and update the figure caption with source information. If applicable, please specify in the figure caption text when a figure is similar but not identical to the original image and is therefore for illustrative purposes only.

The following resources for replacing copyrighted map figures may be helpful:

USGS National Map Viewer (public domain): http://viewer.nationalmap.gov/viewer/
The Gateway to Astronaut Photography of Earth (public domain): http://eol.jsc.nasa.gov/sseop/clickmap/
Maps at the CIA (public domain): https://www.cia.gov/library/publications/the-world-factbook/index.html and [https://www.cia.gov/library/publications/cia-maps-publications/index.html](https://track.editorialmanager.com/CL0/https:%2F%2Fwww.cia.gov%2Flibrary%2Fpublications%2Fcia-maps-publications%2Findex.html/1/010f0198a877b1a4-38679ca3-e103-4d0d-8625-448643e1b603-000000/jQl7KPhQXB1pFCQin1S8VhxR3zYY2-PXeTAjCcx3OrU=224)
NASA Earth Observatory (public domain): http://earthobservatory.nasa.gov/
Landsat: http://landsat.visibleearth.nasa.gov/
USGS EROS (Earth Resources Observatory and Science (EROS) Center) (public domain): http://eros.usgs.gov/#
Natural Earth (public domain): <http://www.naturalearthdata.com/>

**Response:** Thank you for the elaborate guidance on images that may be copyrighted. We want to clarify that the image in Supplemental figure 1 was generated in QGIS. The following publicly available shapefiles were used to generate the image:

- Kenya and Kenyan counties outline obtained from <https://data.humdata.org/dataset/cod-ab-ken> under a Creative Commons Attribution for Intergovernmental Organisations (CC BY-IGO) license.
- Siaya county outline obtained from <https://data.humdata.org/dataset/47-counties-of-kenya> used under a Creative Commons Attribution International (CC BY) license.
- The cluster outlines were produced using mapping data derived as part of study activities.

12. We notice that your supplementary figures are uploaded with the file type 'Figure'. Please amend the file type to 'Supporting Information'. Please ensure that each Supporting Information file has a legend listed in the manuscript after the references list.

**Response:** We have amended the file type for all supplementary figures from Figure to Supporting Information. In addition, we have provided legends for each Supporting Information file, which are now listed in the manuscript after the reference section, as per the journal’s requirements. **(Lines 757-771).**

13. Please include your tables as part of your main manuscript and remove the individual files. Please note that supplementary tables be uploaded as separate "supporting information" files.

**Response:** Thank you for your comments. We want to clarify that the manuscript does not have results presented as tables within the main text. However, supplementary tables are available and have been uploaded separately as “Supporting Information” files, as requested **(Lines 774-777).**

14. Please remove your figures from within your manuscript file, leaving only the individual TIFF/EPS image files, uploaded separately. These will be automatically included in the reviewers’ PDF.

**Response:** Thank you for your guidance on this. We have ensured that the figures have been removed from within the manuscript and uploaded separately with the attachments in the submission form

15. Please include captions for your Supporting Information files at the end of your manuscript, and update any in-text citations to match accordingly. Please see our Supporting Information guidelines for more information: http://journals.plos.org/plosone/s/supporting-information.

If the reviewer comments include a recommendation to cite specific previously published works, please review and evaluate these publications to determine whether they are relevant and should be cited. There is no requirement to cite these works unless the editor has indicated otherwise. 

Please review your reference list to ensure that it is complete and correct. If you have cited papers that have been retracted, please include the rationale for doing so in the manuscript text, or remove these references and replace them with relevant current references. Any changes to the reference list should be mentioned in the rebuttal letter that accompanies your revised manuscript. If you need to cite a retracted article, indicate the article’s retracted status in the References list and also include a citation and full reference for the retraction notice.

**Response:** We have included captions for the Supporting Information files at the end of the manuscript, and ensured that all related in-text citations have been updated to match accordingly. We have checked and ensured that the references are correct and complete and there are no changes made to the reference list in the revised manuscript.

**Reviewers' comments:**

Reviewer's Responses to Questions

**Comments to the Author**

1. Is the manuscript technically sound, and do the data support the conclusions?

The manuscript must describe a technically sound piece of scientific research with data that supports the conclusions. Experiments must have been conducted rigorously, with appropriate controls, replication, and sample sizes. The conclusions must be drawn appropriately based on the data presented.

Reviewer #1: Yes

Reviewer #2: Yes

Reviewer #3: Yes

2. Has the statistical analysis been performed appropriately and rigorously?

Reviewer #1: Yes

Reviewer #2: Yes

Reviewer #3: Yes

3. Have the authors made all data underlying the findings in their manuscript fully available?

The [PLOS Data policy](http://track.editorialmanager.com/CL0/http:%2F%2Fwww.plosone.org%2Fstatic%2Fpolicies.action%23sharing/1/010f0198a877b1a4-38679ca3-e103-4d0d-8625-448643e1b603-000000/FmTaiQtWr2MpgyqgUFJVuwRKzFusts3oHfo_yL4W-Ag=224) requires authors to make all data underlying the findings described in their manuscript fully available without restriction, with rare exception (please refer to the Data Availability Statement in the manuscript PDF file). The data should be provided as part of the manuscript or its supporting information, or deposited to a public repository. For example, in addition to summary statistics, the data points behind means, medians and variance measures should be available. If there are restrictions on publicly sharing data—e.g. participant privacy or use of data from a third party—those must be specified.

Reviewer #1: Yes

Reviewer #2: Yes

Reviewer #3: Yes

4. Is the manuscript presented in an intelligible fashion and written in standard English?

PLOS ONE does not copyedit accepted manuscripts, so the language in submitted articles must be clear, correct, and unambiguous. Any typographical or grammatical errors should be corrected at revision, so please note any specific errors here.

Reviewer #1: Yes

Reviewer #2: Yes

Reviewer #3: Yes

5. **Review Comments to the Author**

Please use the space provided to explain your answers to the questions above. You may also include additional comments for the author, including concerns about dual publication, research ethics, or publication ethics. (Please upload your review as an attachment if it exceeds 20,000 characters)

**Reviewer #1:** The manuscript is well-structured, scientifically rigorous, and highly relevant to the field of malaria vector control as it reports a robust field study that evaluates the sugar feeding behavior of malaria vectors using Attractive Sugar Baits (ASBs) in western Kenya. The research fills an important gap in understanding vector behavior in regions with abundant natural sugar sources and informs deployment strategies for ATSBs in subsequent large-scale trials. There are few suggestions made in my reveiw comments for consideration, including suggestions of the inclusion of some important data into the manuscript.

**Response:** Thank you for your generous comments, We have endeavored to incorporate all the suggestion’s made in the manuscript

**Reviewer #2**:

 There are typos that need to be corrected before publishing,
Progress of malaria vector control has not stalled since 2015. It is ongoing
Instead of using understand use Assess
Where it is statistically insignificant indicate the statistic result
Exophilic malaria vectors are known

**Responses to Reviewer #2:**

We have carefully reviewed the manuscript and corrected all typographical errors including the following areas:

i). The statement on the progress in malaria has now been revised to indicate that the malaria burden has remained substantial. The new statement now reads as follows: “Although steady progress in malaria vector control has been achieved over the years, the malaria burden remains substantial, underscoring the need for complementary mosquito control tools to further reduce transmission.” (**Lines 76-78**)

ii). Thank you for the suggestion to replace the word “understand” with “Assess”. We have reviewed the entire manuscript and we feel that the use of the word “understand” makes better sense than “Assess”. We have therefore not made this amendment in the manuscript.

iii). We have ensured that the statistical results have been indicated in all areas of the manuscript where there is statistical significance.

iv). We have clarified that this represents shifting behaviour of known mosquitoes to engage in outdoor biting that needs further research (**Lines 135-136)**.

**Reviewer #3 comments:**

**Reviewer #3**: The authors report on a field validation study of Attractive Sugar Baits (ASBs) in western Kenya using uranine dye to measure mosquito feeding rates. While methodologically sound, the manuscript requires revision to address critical gaps and improve clarity.

1. The study predicted sufficient feeding rates for epidemiological impact, yet subsequent trials showed no efficacy. The authors inadequately explain this fundamental disconnect. The manuscript can benefit from detailed review of the modeling assumptions, potential validation methodology limitations, and specific comparison with epidemiological trial outcomes.

**Response:** We acknowledge that the predicted feeding rates suggested potential for epidemiological impact, yet subsequent trials did not demonstrate efficacy. While a detailed comparison of modeling assumptions, validation methodologies, and epidemiological outcomes is beyond the scope of the present manuscript, we have revised the discussion to explicitly acknowledge this disconnect as a limitation in the current study and a basis for the need for further modelling work to fully clarify the relationship between feeding rates and epidemiological impact. (**Lines 550-566**).

2. The crossover design is missing a key period effect analysis to test for differences in outcomes over time, independent of the treatment. This is particularly important due to the use of different ASB versions, seasonal variation, and time-dependent confounders. The statistical analysis should also be adjusted for multiple comparisons

**Response:** During the cross-over period analysis, we have now incorporated period as a fixed effect in the model to account for temporal variation between crossover periods. The updated results are presented in the revised manuscript **(Lines 461-466)**.

A significant period effect was observed for An. gambiae (P = 0.032), but not for *An. funestus* (P = 0.093), demonstrating that the crossover design successfully controlled for temporal confounding. A Bonferroni correction (α = 0.05/2 = 0.025) was applied to account for the two co-primary species comparisons. After adjustment for both period effects and multiple comparisons, the findings remained unchanged: there was no significant difference between 2 and 3 ASBs in dye positivity for either species (An. funestus: Bonferroni-adjusted P = >0.999; An. gambiae: Bonferroni-adjusted P = >0.999). The extended monitoring period analyses were exploratory, conducted to characterise heterogeneity in ATSB feeding patterns across species, locations, and sex. These were not pre-specified hypotheses but rather descriptive comparisons to inform future ATSB deployment strategies. Therefore, we did not apply multiple testing correction to these exploratory analyses. This has also been elaborated on the data analysis sub-section of the manuscript **(Lines 341-398)**.

**Abstract**

Line 30: rephrase

**Response:** This has now been rephased. The new statement now reads as follows: “Although steady progress in malaria vector control has been achieved over the years, the malaria burden remains substantial, underscoring the need for complementary mosquito control tools to further reduce transmission.” (**Lines 30-32**).

Line 54-56: Consider breaking it down for better readability, e.g., "The predominant malaria vector was An. funestus sensu lato (s.l.) with an overall dye feeding rate of 11.2%, followed by An. gambiae s.l. at 3.5%. These corresponded to daily feeding rates of 4.8% and 1.2%, respectively."

**Response:** This section has been re-written for better readability. It now reads as follows: “The predominant malaria vector was *An. funestus sensu lato* (s.l.), which had an overall dye feeding rate of 11.2%. This was followed by *An. gambiae* s.l*.* at 3.5%. These corresponded to daily feeding rates of 4.8% and 1.2%, respectively.” (**Lines 104-106**).

**Introduction**

Line 73 replace “falling” with “lies”.

**Response:** The sentence has been modified for clarity and it now reads “Malaria infected an estimated 263 million people with 597,000 deaths globally in 2023 with the greatest burden occurring in Sub-Saharan Africa” (**Lines 129-130**).

Line 114-115: This sentence is a bit passive rephrase.

**Response:** The sentence has now been re-worded as follows “The outcome of this investigation directly informed the design and implementation of a subsequent epidemiological trial aimed at evaluating the efficacy of ATSBs for malaria control” (**Lines 177-179**).

**Methods**

Line 121: consider replacing “non-clustered” with “non-contiguous” for better clarity.

**Response:** This has been duly amended as suggested. (**Line 189).**

Line 122: Explain the terminology “fried egg” earlier.

**Response:** The section of the manuscript where the terminology “fried egg” was used has now been reworded in order to define it more clearly. It now reads as follows:

“Due to the non-contiguous distribution of compounds in western Kenya, a ‘fried-egg’ design was adopted, in which each selected village (the ‘egg white’) incorporated a ≥0.2 km buffer from its edge, surrounding a central ‘core’ area (the ‘yolk’) where sampling was conducted. This approach minimized potential spillover effects from neighbouring untreated areas” (**Lines 189-192**).

Line 136 replace “which” with “that”.

**Response:** Since We had already used the word “that within the same sentence, replacing the word “which” with “that” would mean using the same word twice in a sentence. We have therefore re-worded the sentence for better clarity as follows: “It is a prototype product lacking the toxic ingredient incorporated into ATSBs.” (**Lines 203-204**).

Line 145: rephrase “was maintained” with a more active phrase such as “was used”.

**Response:** The word “was maintained” has been replaced with “was used” in the revised manuscript. (**Line 221**).

Line 159-161: The sentence is a little clunky please rephrase.

**Response:** The statement has been modified as follows: In the two-ASB arm, the bait stations were placed on opposite walls of the structure, while in the three-ASB arm, one device was placed on each of any three walls of the structure. (**Lines 235-189**).

**Discussion**

Line 425: Consider including in the methods and results the laboratory comparison of the two versions of ASBs.

**Response:** The statement has been replaced with the following sentence “Indeed, variation in bait attractiveness between versions has been documented in semi-field comparisons which reinforces the possibility that version changes introduced systematic bias in comparing arms” and a reference has been added to qualify the statement. (**Lines 603-605**).

6. PLOS authors have the option to publish the peer review history of their article ([what does this mean?](https://track.editorialmanager.com/CL0/https:%2F%2Fjournals.plos.org%2Fplosone%2Fs%2Feditorial-and-peer-review-process%23loc-peer-review-history/1/010f0198a877b1a4-38679ca3-e103-4d0d-8625-448643e1b603-000000/cBKsAIeGyHyH-oBNs9TdWYwYIRwV-zKJ3-Gm6jWBiLE=224)). If published, this will include your full peer review and any attached files.

If you choose “no”, your identity will remain anonymous but your review may still be made public.

**Do you want your identity to be public for this peer review?** For information about this choice, including consent withdrawal, please see our [Privacy Policy](https://track.editorialmanager.com/CL0/https:%2F%2Fwww.plos.org%2Fprivacy-policy/1/010f0198a877b1a4-38679ca3-e103-4d0d-8625-448643e1b603-000000/i6MMZ0fzTVwNvCu92N8zj7mn0canJCudZG0lhijttFY=224).

Reviewer #1: No

Reviewer #2: No

Reviewer #3: No

[NOTE: If reviewer comments were submitted as an attachment file, they will be attached to this email and accessible via the submission site. Please log into your account, locate the manuscript record, and check for the action link "View Attachments". If this link does not appear, there are no attachment files.]

While revising your submission, please upload your figure files to the Preflight Analysis and Conversion Engine (PACE) digital diagnostic tool, [https://pacev2.apexcovantage.com/](https://track.editorialmanager.com/CL0/https:%2F%2Fpacev2.apexcovantage.com%2F/1/010f0198a877b1a4-38679ca3-e103-4d0d-8625-448643e1b603-000000/CMNQ2CVcV-oI7BHEjRfCpY6E5K2WQQerOvrJUzXd8II=224). PACE helps ensure that figures meet PLOS requirements. To use PACE, you must first register as a user. Registration is free. Then, login and navigate to the UPLOAD tab, where you will find detailed instructions on how to use the tool. If you encounter any issues or have any questions when using PACE, please email PLOS at [figures@plos.org](mailto:figures@plos.org). Please note that Supporting Information files do not need this step.

**Reviewer #4 Comments:**

**General:** The manuscript is well-structured, scientifically rigorous, and highly relevant to the field of malaria vector control as it reports a robust field study that evaluates the sugar feeding behavior of malaria vectors using Attractive Sugar Baits (ASBs) in western Kenya. The research fills an important gap in understanding vector behavior in regions with abundant natural sugar sources and informs deployment strategies for ATSBs in subsequent large-scale trials. I have some questions and suggestions below:

1. Lines 85–86: The statement 'Attractive targeted sugar baits (ATSBs) are a candidate vector control tool that takes advantage of mosquito sugar feeding behavior' feels incomplete. I suggest expanding it to include a brief explanation of how ATSBs/ASBs work—specifically, how they attract mosquitoes and deliver a toxicant through sugar ingestion—as part of the introduction.

**Response:** We have revised the introduction section of the manuscript to provide a more complete explanation of how ATSBs function. Specifically, we now added the following sentence:

“They consist of a sugar solution laced with an oral toxin and attractants, designed to lure both male and female mosquitoes to feed and ingest the toxicant, thereby reducing local vector populations.” **(Lines 144-146).**

2. Lines 286–295: ASB stations were monitored and replaced throughout the study. This is a key aspect of the study design, and it would be valuable to present this information in table form, showing the durability of the stations over the entire study period. Durability is a standard parameter in ITN bioefficacy monitoring and should likewise be reported for ASB stations.

**Response:** We are grateful for this suggestion and perfectly agree with the reviewer. However, the assessment of the durability of ATSBs is beyond the scope of this manuscript and that information will be published in related studies in future.

3. Lines 324–325 state that “significantly higher dye positivity rates were observed in males compared to females for both An. funestus and An. gambiae.” However, since **only female Anopheles mosquitoes transmit malaria**, the **implication of higher male feeding rates on the overall effectiveness of ASBs**—and ultimately on malaria control—remains unclear. **Please elaborate in the Discussion section** on how this finding affects the interpretation of ASB efficacy.

**Response:** We have made additional points in the discussion section to support the implication of higher dye feeding rates among male mosquitoes. We have now added the following sentence:

**“**Although only female *Anopheles* mosquitoes transmit malaria, high male feeding rates still play a critical role in supporting overall malaria vector control. Males make up a substantial portion of the mosquito population, and their mortality contributes to reducing population size and mating potential, thereby indirectly limiting transmission (Müller et al., 2010; Beier et al., 2012). The higher male feeding observed in our study may also serve as an indicator of bait attractiveness. However, maximizing female exposure remains critical since epidemiological impact depends primarily on reducing female survival and vectorial capacity (Fiorenzano et al., 2017).” **(Lines 536-542).**

4. The authors reported in the Results section in line 331-334 that the overall daily feeding rate on ASBs was estimated at 4.8% for An. funestus and 1.2% for An. gambiae. However, the methods used to obtain these estimates are not described in the Methods section. Would the authors consider including a description of these methods in the Methods section?

**Response:** A section on “**Conversion of dye-positivity to daily feeding rates” has been added to the manuscript. (Lines 328-336).**

5. The authors reported the Relationship of bait station density and rainfall to dye positivity in from line 340. These are not described anywhere in the Methods section. Would the authors please do so?

**Response: We have ensured that the information about rainfall and ASB comparisons are available in Lines 197-200 and B 391-397 of the methods sections.**

6. Sporozite detection has been mention in line 272-275. I understand that this was not the main call of this paper but it is important to include a supplementary table showing these results.

**Response:** While we acknowledge the importance of sporozoite detection, this outcome was not a primary focus of the present study. We believe that the summary results reported in the manuscript adequately capture the sporozoite rates observed and provide sufficient context without the need for an additional supplementary table.

7. The conclusion in line 429 does not address the objectives of this study. Please revise it to include the key findings in relation to the stated objectives.

**Response:** We have reformated

We once again thank you and the reviewers for the valuable feedback that has helped us improve the quality and clarity of our manuscript. We believe the revisions have strengthened the paper, and we remain happy to address any further questions or clarifications that may arise.

Sincerely

Jackline Jeruto Kosgei.
